# Quality Control Standards for the Roots of Three *Plumbago* Species

**DOI:** 10.4103/0250-474X.62254

**Published:** 2010

**Authors:** S. Ariyanathan, A. Saraswathy, G. V. Rajamanickam

**Affiliations:** Centre for Advanced Research in Indian system of Medicine (CARISM), SASTRA University, Thanjavur-613 402, India; * Captain Srinivasa Murti Drug research Institute for Ayurveda and Siddha (CCRAS), Anna Hospital Campus, Arumbakkam, Chennai-600 106, India

**Keywords:** *P. rosea*, *P. zeylanica*, plumbagin, *Plumbago capensis*, quality control parameters, Root

## Abstract

Physicochemical parameters of roots of three *Plumbago* species, *Plumbago capensis*, *P. rosea* and *P. zeylanica* belonging to Plumbaginaceae were analyzed. Microbial contamination, aflatoxins, pesticide residue and heavy metal content were also determined. Attempt has also been made to estimate the biologically active chemical plumbagin present in them and the data compared. The study ensures that the quality control parameters do help in the proper standardization of the crude drugs in drug development process for global acceptance.

Standardization of raw drugs in herbal industry is an important step towards quality control. Several analytical parameters such as physico–chemical constants, estimation of elements, heavy metals, microbial contamination, aflatoxins and pesticide residue are to be carried out as a measure of quality check. The World Health Organization[1] emphasized certain quality standards and has proposed certain guidelines for the assessment and development of standard herbal products. The plant *Plumbago zeylanica* of Plumbaginaceae known as *Chitraka/Chitramoolam* is a popular drug in Ayurveda and Siddha[1]. It finds place in several of the compound formulations of these systems of medicine, as one of the ingredients[2,3]. In times of scarcity of *P. zeylanica* roots, the roots of the related species namely *P. capensis* and *P. rosea,* which are indigenous to India are often used. The roots of three species are recognized due to the presence of naphthaquinones namely plumbagin and its derivatives. The roots of *P. rosea* and *P. zeylanica* have been evaluated for activities[4,5]. The paper deals with the physico-chemical standards such as loss on drying at 105°, ash, acid-insoluble ash, water soluble ash, crude fibre content, alcohol soluble extractive, water soluble extractive, analysis of heavy metals and other elements, pesticide residues, aflatoxins, microbial contamination and assay of plumbagin through HPTLC for the roots of three *Plumbago* species.

## MATERIALS AND METHODS

Roots of *P. capensis* were procured from the local market at Nagercoil, Tamil Nadu. Roots of *P. rosea* and *P. zeylanica* were collected from Erode and Arakonam near Chennai, Tamil Nadu, respectively. The crude drugs were identified at Captain Srinivasa Murti Drug Research Institute for Ayurveda and Siddha (CCRAS), Arumbakkam, Chennai, India. Voucher specimens of *P. capensis* (00583), *P. rosea* (00502) and *P. zeylanica* (00518) have been deposited in the herbarium of this institute.

The procedures recommended in WHO guidelines[6], Indian Pharmacopoeia[7] and AOAC[8] were followed to determine loss on drying at 105°, total ash, water-soluble ash, acid-insoluble ash, alcohol soluble, water soluble extractive contents, analysis of heavy metals and other elements, aflatoxins, pesticide residues.

The roots of the plants were given a quick wash with water, shade dried and coarsely powdered. The powdered samples were weighed (each 1 g) into separate conical flasks and treated with 5 ml of concentrated nitric acid. The flasks were covered with watch glasses and heated for an hour; the contents of the flasks were treated with additional 5 ml of nitric acid, followed by 2 ml of 30 % hydrogen peroxide solution. The heating was continued till the clear solution was obtained. The mixture was diluted with deionised water and filtered through Whatman No. 42 filter paper and the solutions were made up to 50 ml[9].

Aflatoxins were determined by Kobra cell technique using Agilent HPLC instrument as per the method ASTA[10]. Pesticide residues were analyzed using GC-MS Agilent instrument equipped with mass selective detector as per the method AOAC[8]. For determination of microbial load, 1g of each sample was weighed accurately in separate flasks and 99 ml of sterile distilled water was added. The samples in the flask were kept in a mechanical shaker for few minutes to obtain uniform suspension of microorganisms. The dilution is 1:100 or 10^-2^ from, which 1 ml was transferred to 9 ml of sterilized distilled water to make a 1:1000 dilution and this procedure was repeated up to 10^-6^ dilution. Each 0.1 ml of serially diluted sample was inoculated to the sterile plates containing nutrient agar, SS agar and potato dextrose agar (PDA) by spread plate method. Nutrient agar and SS agar plates were incubated at 37° for 24 h and PDA plates were incubated at room temperature for 3-5 days. Bacterial and fungal colonies were counted using a colony counter[6].

For the estimation of plumbagin, 1 g of each of coarsely powdered root samples of *P. capensis, P. rosea* and *P. zeylanica* were weighed accurately and extracted exhaustively with chloroform (50 ml) in a Soxhlet apparatus. The chloroform extract was filtered, concentrated and made upto 5 ml in volumetric flask[11–15]. The standard solution was prepared by dissolving 10 mg of plumbagin in 10 ml of chloroform. From this stock solution, 1 ml was pipetted out into a 10 ml standard flask and made up to the mark. Sample solution of the roots of *P. capensis* 5 μl, *P. rosea* 5 μl and *P. zeylanica* 10 μl and different concentrations of standard solution varying from 2 to 10 μl of plumbagin were applied on aluminium plate precoated with silica gel 60F_254_ 0.2 mm thickness on different tracks as 6 mm bands by a Camag Linomat applicator V. The plate was developed in a twin trough glass chamber pre-saturated with toluene:ethyl acetate (4:1) and allowed to run upto a distance of 8 cm. After air drying, the plate was visualized under UV at λ 254 and 366 nm and scanned using scanner 3. Calibration graph was obtained with plumbagin ranging from 200 to 1000 ng (correlation coefficient, r= 0.99946).

## RESULTS AND DISCUSSION

Table 1 summarizes the various physico-chemical constants observed for the roots of three species. The physico-chemical analysis indicated the ash content of 2.62% in *P. capensis*, 8.01% *in P. rosea* and 3.1% *in P. zeylanica*, respectively which is due to the presence of inorganic matter present in these *Plumbago* species. Inorganic matter was found to be higher in *P. rosea* than *P. zeylanica*, whereas it was found to be less in *P. capensis*. Acid-insoluble ash indicates the presence of more siliceous matter in the drug. It was found to be 0.68% in *P. capensis*, 1.16% in *P. rosea* and 0.96% in *P. zeylanica*. More siliceous matter was found in *P. rosea* than *P. zeylanica* and it was noticed to be less in *P. capensis*. The alcohol soluble extractive reveals the presence of polar compounds like anthraquinones, alkaloids, glycoside of flavonoids, steroids and triterpenoids present in the plant materials. It was seen to be 9.14% in *P. capensis*, 5.57% in *P. rosea* and 12.83% in *P. zeylanica*. The water soluble extractive reveals the presence of water soluble matters such as sugars, carboxylic acids, vitamins and amino acids and it was found to be 14.67% in *P. zeylanica*, 13.18% in *P. capensis* and 5.57% in *P. rosea*. Both the alcohol soluble extractive and water soluble extractive are higher in *P. zeylanica,* followed by *P. capensis* and *P. rosea (P. zeylanica>P. capensis>P. rosea*). Loss on drying at 105° is determined since the presence of excess moisture is conducive to the promotion of mould and bacterial growth, and subsequently to deterioration and spoilage of the drug. The moisture content was calculated to be 8.66% in *P. rosea*, 8.18% in *P. zeylanica* and 8.11% in *P. capensis* (*P. rosea>P. zeylanica>P. capensis*). Crude fibre consists largely of cellulose and lignin (97%) plus some mineral matter. It represents only 60 to 80% of cellulose and 4 to 6% of lignin. It is useful as a measure of nutritive value of animal feeds and also in the analysis of various foods and food products to detect adulteration, quality and quantity. The fibre content was accounted to be 14.30% in *P. zeylanica*, 12.51% in *P. capensis* and 9.79% in *P. rosea* (*P. zeylanica>P. capensis>P. rosea*). The data evolved can be considered for laying down the pharmacopoeial standards for the roots of the three *Plumbago* species.

**TABLE 1:** PHYSICO-CHEMICAL VALUES FOR *PLUMBAGO* SPECIES

Parameter	*P. capensis* (% w/w)	*P. rosea* (% w/w)	*P. zeylanica* (% w/w)
Loss on drying at 105^°^	8.11	8.66	8.18
Total Ash	2.62	8.01	3.11
Water-soluble ash	1.89	6.01	2.27
Acid-insoluble ash	0.68	1.16	0.96
Alkalinity of water soluble ash	0.2 ml/g	0.3 ml/g	0.2 ml/g
Alcohol-soluble extractive	9.14	5.57	12.83
Water-soluble extractive	13.18	10.85	14.67
Crude fibre content	12.51	9.79	14.30

Values are mean of 3 readings

The different inorganic elemental content present in the plant part studied are shown in Table 2. Iron is the most indispensable metal to humans and its deficiency is characterized by anemia. The root of *P. rosea* contained significant amount of iron which is diuretic, demulcent and useful in the treatment of dysentery and croup[16]. Copper deficiency is characterized by chronic or recurrent diarrhea, low resistance to infection and anemia[17,18]. The maximum concentration of copper was found in *P. zeylanica*, which may be used to cure anemia, rheumatism and emetic conditions. Manganese is essential for haemoglobin formation. It is essential for growth, reproduction and skeleton development in humans[19,20]. The maximum concentration of manganese was observed in *P. zeylanica>P. rosea>P. capensis*, which are used in skin diseases, scabies, piles and rheumatism[21,22]. Zinc is a component of many metalloenzymes and also a membrane stabilizer and a stimulator of the immune response[23,24]. Its deficiency leads to loss of appetite, and impaired immune function. In more severe cases, zinc deficiency causes hair loss, diarrhea, delayed sexual maturation, impotence, hypogonadism in males, and eye and skin lesions[25,26]. The zinc content was high in *P. zeylanica* while the amount is almost equal in *P. capensis* and *P. rosea*, which may be useful in skin diseases and rheumatism.

**TABLE 2:** INORGANIC MINERAL ANALYSIS OF *PLUMBAGO* SPECIES

Sample	Fe	Cu	Mn	Ni	Zn	Co	Cr	Na	K	Ca
*P. capensis*	5.86	0.42	0.55	0.03	0.34	0.06	0.02	94	89	42
*P. rosea*	6.47	0.22	0.92	0.01	0.34	0.06	0.00	82	113	18
*P. zeylanica*	2.92	0.47	1.17	0.02	0.51	0.09	0.00	80	89	24

Values in ppm

Chromium plays a vital role in metabolism of carbohydrates and its deficiency leads to diabetes in human body. Chromium deficiency leads to hyperglycemia, growth failure, cataract and arteriosclerosis[27–29]. The maximum content of chromium was seen in *P. capensis* followed by *P. zeylanica* and *P. rosea* which justified the use of the root in Ayurveda and Siddha systems of medicines for diabetes.

Sodium is involved in intracellular and extracellular fluid balance and the maintenance of the viscosity of blood[29,30]. Sodium plays an important role in the transport of metabolites. The maximum content of Sodium was seen in *P. capensis* followed by *P. rosea* and *P. zeylanica*. Potassium is a diuretic. Both sodium and potassium take part in ionic balance of the human body and maintain tissue excitability[29]. The maximum content of Potassium was seen in *P. rosea.* Calcium plays an important part in nerve-impulse transmission and in the mechanism of neuromuscular system[29]. Calcium content was in the order of *P. capensis>P. zeylanica>P. rosea* (Table 2).

**Fig. 1:**
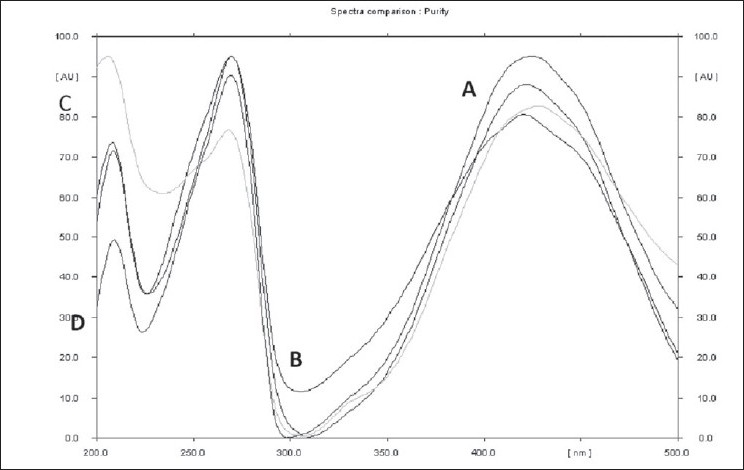
UV spectrum showing superimposition of plumbagin Superimposable UV spectra of plumbagin and chloroform extracts of three *Plumbago* species. A. P. *capensis*, B. P. *rosea*, C. P. *zeylanica* and D. plumbagin

**Fig. 2:**
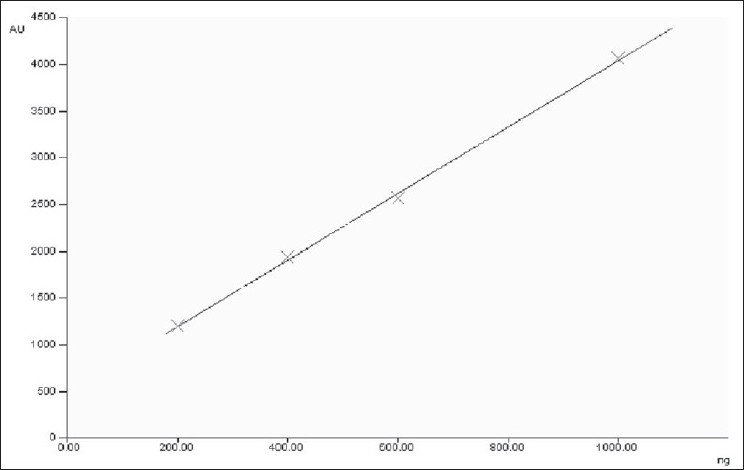
Linearity curve Plumbagin and chloroform extracts of three *Plumbago* species

**Fig. 3:**
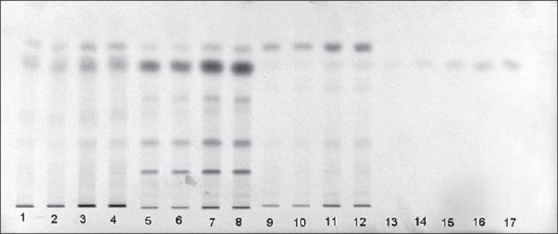
TLC chromatogram viewed at 254 nm Plumbagin and chloroform extracts of three *Plumbago* species under UV 254 nm. Tracks 1-4, *P. capensis*, tracks 5-8, *P. rosea*, tracks 9-12, *P. zeylanica* and tracks 13-17, plumbagin

**Fig. 4:**
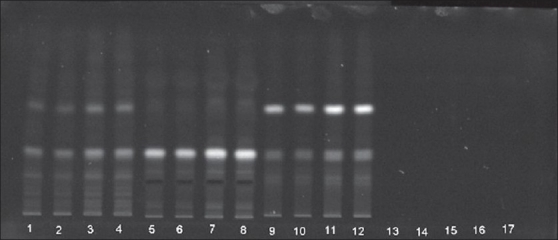
TLC chromatogram viewed at 366 nm Plumbagin and chloroform extracts of three *Plumbago* species under UV 366 nm. Tracks 1-4, *P. capensis*, tracks 5-8, *P. rosea*, tracks 9-12, *P. zeylanica* and tracks 13-17, plumbagin

The contents of heavy metals namely lead, mercury, cadmium and arsenic are found to be within the permissible limit[31] for the three *Plumbago* species, indicating that the three roots are safe to utilize as drugs (Table 3). The report of analysis for aflatoxins in the three *Plumbago* species is given in the Table 4. The aflatoxins B_1_, B_2_, G_1_, and G_2_ were below the detecting level revealing that they are free from toxins and are safe for internal use. Further the studies indicated that the absence of these aflatoxins would help to increase in shelf life of the raw drug. The various pesticide residues such as α,β,γ,δ-hexachlorocyclohexane (α,β,γ,δ-HCH), o,p'-dichlorodiphenyl trichloroethane(o,p-DDT), p,p'-dichlorodiphenyltrichloroethane (p,p'-DDT), o,p-dichlorodiphenyl dichloroethylene (o,p-DDD), p,p-dichlorodiphenyldichloroethylene (p,p-DDD), α-endosulphan, β-endosulphan, o,p-dichlorodiphenyldichloroethane (o,p-DDE) and p,p'-dichlorodiphenyldichloroethane (p,p-DDE) are analyzed. None of the above pesticides is detected (DL: 0.01 ppb) in the three *Plumbago* species, indicating that they are safe for their usage as drugs (Table 5). Analysis for the microbial load for the three *Plumbago* species is found to be within the limit of WHO guidelines, indicating that they are free from pathogens and can be used as drugs (Table 6). TLC studies revealed that the solvent system toluene:ethyl acetate (4:1) was ideal and gave a single spot with R_f_ 0.70 for plumbagin and well resolved spots for the test samples. The identity of plumbagin in the samples was shown by superimposable UV spectra (fig. 1) at R_f_ 0.70 in the sample solutions. The peak purity data is given in Table 7. The linear regression curve of plumbagin was obtained for the amount of 200 to 1000 ng. The sample solutions also showed plumbagin within this curve as shown in fig. 2. The spots of the chromatogram were visualized under UV at λ 254 and 366 nm were shown in figs. 3 and 4. The amount of plumbagin was calculated from the linearity curve. The percent content of plumbagin was found to be in the decreasing order of *P. rosea* (0.17), *P. capensis* (0.04) and *P. zeylanica* (0.01). The physicochemical parameters evaluated are useful in standardization of the roots of the three species. Heavy metals, aflatoxins, pesticidal residue and microbial load for the three *Plumbago* species are found to be within the limit of WHO guidelines, indicating that they are free from pathogens and they are safe to be used in ISM. The data obtained from the study would be useful in the identification of the roots of these *Plumbago* species and serve as standards. Quantification of the marker compound, plumbagin done by HPTLC is found to be in the order of *P. rosea>P. capensis>P. zeylanica,* which can also be considered as an additional parameter for quality check of these three roots. *P. rosea* is found to contain the highest (0.17% w/w) of plumbagin. The data evolved can be considered for laying down the pharmacopoeial standards for the roots of the three *Plumbago* species.

**TABLE 3:** HEAVY METAL ANALYSIS OF *PLUMBAGO* SPECIES

Element	*P. capensis*	*P. rosea*	*P. zeylanica*	Permissible Limits
As	0.0002	0.0001	0.0001	3
Cd	0.0028	0.0007	0.0065	0.3
Pb	0.2359	0.3116	0.2605	10
Hg	0.0037	0.0053	0.0031	1

Values in ppm. Permissible limits are as per the Ayurvedic Pharmacopoeia of India, 2008

**TABLE 4:** AFLATOXINS IN THREE *PLUMBAGO* SPECIES

Aflatoxins	*P. capensis*	*P. rosea*	*P. zeylanica*
Aflatoxin B_1_	BDL (DL:1.0 ppb)	BDL (DL:1.0 ppb)	BDL (DL:1.0 ppb)
Aflatoxin B_2_	BDL (DL 0.5 ppb)	BDL (DL 0.5 ppb)	BDL (DL 0.5 ppb)
Aflatoxin G_1_	BDL (DL 1.0 ppb)	BDL (DL 1.0 ppb)	BDL (DL 1.0 ppb)
Aflatoxin G_2_	BDL (DL 0.5 ppb)	BDL (DL 0.5 ppb)	BDL (DL 0.5 ppb)

BDL denotes below detectable limit and DL detectable limit

**TABLE 5:** PESTICIDAL RESIDUE ANALYSIS OF THREE *PLUMBAGO* SPECIES

Pesticides	*P. capensis*	*P. rosea*	*P. zeylanica*
α - HCH	ND	ND	ND
β - HCH	ND	ND	ND
γ - HCH	ND	ND	ND
δ -HCH	ND	ND	ND
*op*-DDT	ND	ND	ND
*pp*-DDT	ND	ND	ND
*op*-DDE	ND	ND	ND
α- Endosulfan	ND	ND	ND
β - Endosulfan	ND	ND	ND
*op*-DDD	ND	ND	ND
*pp*-DDD	ND	ND	ND

Detection limit is 0.01 ppm, ND denotes not detectable and DL detectable limit

**TABLE 6:** DETERMINATION OF MICROBIAL LOAD FOR THREE *PLUMBAGO* SPECIES

Name of bacteria	CFU			WHO limit	Inference
			
	*P. capensis*	*P. rosea*	*P. zeylanica*		
*Eschericha coli*	2×10^1^	8	-	10^2^	Within the limit
*Salmonella* sp.	-	-	-	Absence	complies
*Shigella* sp.	-	-	-	Absence	complies
*Enterobacter* sp.	-	-	-	10^4^	complies
Total bacterial count	12×10^2^	18×10^3^	9×10^2^	10^7^	Within the limit
Yeast & Mould	1×10^2^	10×10^1^	13×10^2^	10^4^	Within the limit

CFU denotes colony forming unit

**TABLE 7:** PEAK PURITY TEST

Sample	R_f_	r(s,m)	r(m,e)
*P. capensis*	0.69	0.9998	0.9992
*P. rosea*	0.68	0.9999	0.9996
*P. zeylanica*	0.69	0.9999	0.9998
Plumbagin	0.70	0.9999	0.9996
